# Daily tobacco use and problem drinking among urban adults in South Africa: a longitudinal study

**DOI:** 10.11604/pamj.2019.32.51.17256

**Published:** 2019-01-29

**Authors:** Karl Peltzer, Li-Wei Chao, Shandir Ramlagan, Helena Szrek

**Affiliations:** 1 HIV/AIDS/STIs and TB Research Programme, Human Sciences Research Council, Pretoria, South Africa; 2 Department of Research and Innovation, University of Limpopo, Turfloop, South Africa; 3 University of Porto, Porto Business School, Porto, Portugal; 4 University of Porto, Center for Economics and Finance (cefUP), Porto, Portugal; 5 University of Pennsylvania, Population Studies Center and Leonard Davis Institute of Health Economics, Philadelphia, USA

**Keywords:** Daily tobacco use, problem drinking, health risk behaviour, urban dwellers, South Africa

## Abstract

**Introduction:**

There is a lack of longitudinal studies investigating daily tobacco use and problem drinking in Africa. The aim of this study was to explore the prevalence of daily tobacco use and problem drinking and to determine the factors associated with daily tobacco use and problem drinking among urban dwellers in a longitudinal study in South Africa.

**Methods:**

Electronic interview data were collected from 2213 adults (mean age 45.7 years, SD=15.1; range 20-97) at time 1 (baseline assessment) and Time 2 (12 months follow-up assessment) from one urban centre in South Africa.

**Results:**

Daily tobacco use only, was at time 1 24.0% and at time 2 23.4%, a decrease of 0.5%. Problem drinking only was at time 1 19.6% and at time 2 21.1%, an increase of 1.5%. Concurrent daily tobacco use and problem drinking increased from time 1 9.5% to 10.3% at time 2, an increase of 0.8%. In longitudinal regression analyses, being male and being born in current city were significantly associated with all three substance use indicators (daily tobacco use; problem drinking; and concurrent daily tobacco use and problem drinking). In addition, older age, not currently married, lower education, underweight and higher levels of perceived stress were associated with daily tobacco use and younger age was associated with problem drinking.

**Conclusion:**

High prevalence of daily tobacco use and problem drinking were found among urban dwellers and several socio-demographic (being male, being born in the city, not married and lower education) and health variables (being underweight and perceived stress) were identified which can guide substance use intervention programmes for this population.

## Introduction

In the "South African National Health and Nutrition Examination Survey", conducted in 2012, of adult South Africans, 9.6% were engaged in concurrent current tobacco use and problem drinking, 20.3% in problem drinking only and 18.2% in current tobacco use only [1]. Various studies have shown that the concurrent use of alcohol and tobacco is more detrimental to health than each drug on its own [2-4]. There is a lack of studies in Africa investigating problem drinking and daily tobacco use over time in a longitudinal study. Evaluating tobacco use and drinking change is of "importance to epidemiologic studies because it is often the persistence of lifestyle behaviours, such as smoking and drinking, that influences health" [5]. As reviewed by Phaswana-Mafuya *et al*[1], risk factors for concurrent alcohol and tobacco use have been identified as sociodemographic variables (lower education, male gender, younger age, lower socioeconomic status) and psychosocial (drug use, poor mental health). Urban populations may be at greater risk of daily tobacco use and problem drinking in South Africa [1, 6-8]. Therefore, the aim of this study was to explore the prevalence of daily tobacco use and problem drinking and its determinants among urban dwellers in a longitudinal study in South Africa.

## Methods

**Sample and procedure:** The sample included second wave (N = 2213, in 2012) and third wave (N = 2213, in 2014) of the "South African Panel Study of Small Business and Health, a longitudinal survey in African townships in South Africa that collects data on health, psychology, and entrepreneurship from owners of small businesses and from randomly selected respondents who do not own businesses" [9]. A two stage stratified probability sampling design was used to select the full sample from twenty-eight African dominated enumeration areas in the Tshwane Municipality [9]. The interviews were conducted in the preferred language of the respondent, “using Google Phones with Android 1.5 operating system and interview software Open Data Kit (ODK) Collect version 1.1 [9]. Study participants signed informed consents and the protocol was approved by participating institution's ethics review boards.

**Measures:** *Tobacco use* was assessed with four questions: 1) Do you currently smoke any tobacco products, such as cigarettes, cigars or pipes? If they answered yes, they were asked 2) Do you currently smoke tobacco products daily? 3) Do you currently use any smokeless tobacco, such as snuff, chewing tobacco, betel? If they answered yes, they were asked 4) Do you currently use smokeless tobacco products daily? [10]. Daily tobacco use included the current daily use of smoking tobacco products and/or current daily use of smokeless tobacco products. *Hazardous or harmful alcohol use (or problem drinking)* defined with a cut-off score of four for men and three for women on the Alcohol Use Disorder Identification Test (AUDIT)-C [11]. The Cronbach alpha for the AUDIT-C was 0.81 for this sample.

**Concurrent daily tobacco use and problem drinking:** Based on the above measures on daily tobacco use and problem drinking, concurrent was defined as using both tobacco daily and problem drinking, not at exactly the same time but within a brief time period. The general health status was measured using SF-12, an instrument with 12 items that reflect eight sub-domains, such as physical functioning (2 items, Cronbach α: 0.81) and mental health (2 items, α: 0.82). For each participant, the SF-12 scoring algorithm generates a Physical health Component Summary (PCS-12) score and a Mental health Component Summary (MCS-12) score, with lower scores indicating higher activity limitations [12]. *Psychological distress* was assessed with the 10-item "Kessler Psychological Distress scale" (K-10) [13]. (Cronbach alpha 0.78). The participant's feelings of stress is measured by the *Perceived Stress Scale* (PSS-4) [14]. (Cronbach alpha 0.63). *Sociodemographic factors* that included gender, age, marital status, education, income and born in current city or not, were also assessed. *Body mass index (BMI)* was calculated as "weight in kg divided by height in metre squared" [15], using the participant's weight and height that were measured by trained research nurses. A list of ever-diagnosed *chronic conditions* was also included as control variables and these included migraine headache, lower back pain, hypertension, stomach ulcer, arthritis, heart attack or angina, diabetes, emphysema/bronchitis, asthma, cancer, epilepsy and stroke.

**Data analysis:** Data analysis was conducted using STATA software version 15.0 (Stata Corporation, College Station, Texas, USA). Frequencies, means, and standard deviations were calculated to describe the sample. Changes in daily tobacco use, problem drinking, or concurrrent daily tobacco use and problem drinking were calculated and the significance of the difference over time was tested using the Wilcoxon matched pairs signed-ranks test, using a significance level at 10%. The association between sociodemographic factors, health variables and daily tobacco use only, problem drinking only and concurrent daily tobacco use and problem drinking was modelled using logistic regression with generalised estimating equations (GEE) to account for repeated measures. This analysis provides estimates of the average effects over the two surveys accounting for within-person correlation in the two surveys, reporting Odds Ratios and 95% Confidence Intervals. Time dependent variables measured at each survey included daily tobacco use, problem drinking, concurrent daily tobacco use and problem drinking, physical health (PCS), mental health (MCS), psychological distress and perceived stress. Sociodemographic variables (age, gender, income, education, marital status) and body weight status were only available at one time, was also added in the GEE model. Potential multi-collinearity between variables was assessed with variance inflation factors, none of which exceeded the value of 2.0. P < 0.05 was considered significant.

## Results

**Sample characteristics and prevalence rates of tobacco and alcohol use:** The sample included 2213 participants at Time 1 (mean age 44.7 years, SD = 15.08; range 20-95) and 2213 participants at Time 2 (mean age 46.7 years, SD = 15.08; range 22-97). Almost two thirds (62.5%) were female, 35% were 50 years and older, 36.8% had Grade 12 or more education, 54.1% were single, divorced or widowed and 56.4% had been born in the current city they were living in. More than half (56.3%) were overweight or obese and 48% had one or more chronic condition (Table 1).

**Table 1:** Baseline sample characteristics

Variable	Total sample	Daily tobacco use only	Problem drinking only	Daily tobacco use and problem drinking only
	N=2,213 (%)	N=531 (%)	N=434 (%)	N=207 (%)
**Gender**				
Female (0)	1,383 (62.49)	207 (38.98)	159 (36.64)	43 (20.77)
Male (1)	830 (37.51)	324 (61.02)	275 (63.36)	164 (79.23)
Age				
19-35	660(29.82)	116 (21.85)	182 (41.94)	66 (31.8)
36-49	778 (35.16)	172 (32.39)	140 (32.26)	72 (34.78)
50-95	775 (35.02)	243 (45.76)	112 (25.81)	69 (33.33)
**Marital status**				
Married (1)	1,016 (45.91)	238 (44.82)	174 (40.09)	85 (41.06 )
Single, Widowed, Divorced (0)	1,197 (54.09 )	293 (55.18)	260 (59.91)	122 (58.94)
**Education**				
Grade 0-7	530 (23.95)	175 (32.96)	75 (17.28)	47 (22.71)
Grade 8-11	868 (39.22)	228 (42.94)	168 (38.71)	92 (44.44)
Grade 12 or more	815 (36.83)	128 (24.11)	191 (44.01)	68 (32.85)
**Income**				
Low	593 (26.80)	148 ( 27.87)	107 (24.65)	57 ( 27.54)
Medium	925 (41.80)	227 (42.75)	188 (43.32)	82 (39.61)
High	695 (31.41)	156 (29.38)	139 (32.03)	68 (32.85)
**Born in current city**				
No (2)	966 (43.65)	215 (40.49)	139 (32.03)	69 (33.33)
Yes (1)	1,247 (56.35)	316 (59.51)	295 (67.97)	138 (66.67)
BMI				
Under	111 (5.02)	49 (9.23)	36 (8.29)	27 (13.04)
Normal	690 (31.18)	236 (44.44)	198 (45.62)	111 (53.62)
Overweight	533 (24.08)	94 (17.70)	88 (20.28)	32 (15.46)
Obese	714 (32.26)	108 (20.34)	83 (19.12)	21 (10.14)
**Perceived stress**				
Low (0-3)	568 (25.6)	103 (19.40)	107 (24.65)	35 (16.91)
Medium (4-6)	709 (32.04)	164 (30.89)	148 (34.10)	74 (35.75)
High (7-16)	931 (42.07)	264 (49.72)	179 (41.24)	98 (47.34)
Psychological distress (Mean)	18.74728	19.66102	18.80876	19.48792
PCS (Mean)	50.17055	49.24257	51.25931	50.61039
MCS (Mean)	49.37096	48.11281	49.08411	48.14358
**Chronic conditions**				
0	1150 (51.97)	279 (52.54)	249 (57.37)	118 (57.00)
1	572 (25.85)	131 (24.67)	113 (26.04)	53 (25.60)
2	276 (12.47)	67 (12.62)	50 (11.52)	27 (13.04)
3 or more	215 (9.72)	54 (10.17)	22 (5.07)	9 (4.35)

PCS=Physical health Component Summary; MCS=Mental health Component Summary

**Tobacco and alcohol use and other health variables over time:** Daily tobacco use was at time 1 23.99% and at time 2 23.41%, a non-significant decrease of 0.58% (P = 0.323). Problem drinking was at Time 1 19.61% and at time 2 21.1%, an increase of 1.5%, which is significant at the 10% level (z = 1.83 by Wilcoxon signed-ranks test, p = 0.067). Concurrent daily tobacco use and problem drinking has increased from Time 1 9.46% to 10.3% at time 2 (Table 2), an increase of 0.84%, also significant at the 10% level (z = 0.175 by Wilcoxon signed-ranks test, p = 0.080).

**Table 2:** Change in tobacco and alcohol use over 12 months (N = 2213)

Variable	At baseline (N=2213)	At 12 months (N=2213)	Net change from baseline to 12 months	P-value*
	N (%)	N (%)	%	
**Total Sample**				
Daily tobacco use	531 (23.99)	518 (23.41)	-0.58	0.323
Problem drinking	434 (19.61)	467 (21.10)	1.49	0.067
Daily tobacco use and problem drinking	207 ( 9.46)	228 (10.30)	0.84	0.080
**Men**				
Daily tobacco use	324 (39.04)	319 (38.43)	-0.61	0.553
Problem drinking	275 (33.13)	301 (36.27)	3.13	0.060
Daily tobacco use and problem drinking	164 (19.76)	180 (21.69)	1.93	0.124
**Women**				
Daily tobacco use	207 (14.97)	199 (14.39)	-0.58	0.428
Problem drinking	159 (11.50)	166 (12.00 )	-0.50	0.511
Daily tobacco use and problem drinking	43 ( 3.11)	48 (3.47)	0.36	0.396

* p-value: statistical significance in the difference of the means; *P* < 0.05 was considered significant

**Associations with daily tobacco use and problem drinking:** in longitudinal regression analyses, men's odds of daily tobacco use was 2.99 times that of females, 2.71 for problem drinking, and 6.56 for concurrent daily tobacco use and problem drinking. Daily tobacco use increased with age, while problem drinking decreased with age. Daily tobacco users were less educated, more likely to be currently unmarried and were more likely to have been born in the current city. Overweight or obese urban dwellers were less likely to engage in daily tobacco use, but higher perceived stress was associated with daily tobacco use. Problem drinkers were also more likely to have been born in the current city, but not associated with education level. Better mental health (MCS), psychological distress, physical health (PCS) and the presence of chronic conditions had no influence on daily tobacco use, problem drinking and concurrent daily tobacco use and problem drinking (Table 3).

**Table 3:** Logistic regression model with generalized estimating equations (GEE) for the association between sociodemographic and health variables on daily tobacco use, problem drinking and dual tobacco and alcohol use

Variable	Daily tobacco use only	Problem drinking only	Daily tobacco use and problem drinking
	AOR (95% CI)	AOR (95% CI)	AOR (95% CI)
Daily tobacco use	-----	4.002 (3.20 ; 5.01)***	-----
Problem drinking	4.091 (3.28 ; 5.10)***	-----	-----
**Gender**			
Female (0)	Reference	Reference	Reference
Male (1)	2.993 (2.37 ; 3,79)***	2.71 (2.16 ; 3.40)***	6.560 (4.79 ; 8.98)***
**Age**			
19-35	Reference	Reference	Reference
36-49	2.118 (1.58 ; 2.84)***	0.563 (0.34 ; 0.72)***	1.346 (0.95 ; 1.92)
50-95	3.320 (2.34 ; 4.71)***	0.420 (0.31 ; 0.57)***	1.490 (0.94 ; 2.36)
**Marital status**			
Married (1)	0.740 (0.59 ; 0.92)**	0.971 (0.80 ; 1.43)	0.739 (0.56 ; 0.98)*
Single, Widowed, Divorced (0)	Reference	Reference	Reference
**Education**			
Grade 0-7	Reference	Reference	Reference
Grade 8-11	0.707 (0.53 ; 0.95)*	1.049 (0.77 ; 1.43)	1.127 (0.76 ; 1.67)
Grade 12 or more	0.394 (0.28 ; 0.56)***	1.281 (0.91 ; 1.80)	0.886 (0.55 ; 1.41)
**Income**			
Low	Reference	Reference	Reference
Medium	0.931 (0.71 ; 1.22)	1.198 (0.92 ; 1.55)	0.985 (0.70 ; 1.39)
High	0.983 (0.74 ; 1.31)	1.404 (1.07 ; 1.84)*	1.370 (0.96 ; 1.96)
**Born in current city**			
Yes	1.264 (1.00 ; 1.60)	1.572 (1.27 ; 1.94)***	1.383 (1.03 ; 1.85)*
No	Reference	Reference	Reference
**Body Mass Index**			
Under	Reference	Reference	Reference
Normal	0.725 (0.48 ; 1.10)	1.132 (0.74 ; 1.73)	0.767 (0.49 ; 1.21)
Overweight	0.346 (0.22 ; 0.54)***	1.004 (0.64 ; 1.56)	0.373 (0.22 ; 0.63)***
Obese	0.393 (0.25 ; 0.62)***	0.828 (0.52 ; 1.32)	0.310 (0.17 ; 0.55)***
**Perceived stress**			
Low (0-3)	Reference	Reference	Reference
Medium (4-6)	1.366 (1.09 ; 1.72)**	0.948 (0.76 ; 1.18)	1.500 (1.11 ; 2.03)**
High (7-16)	1.648 (1.28 ; 2.12)***	0.870 (0.68 ; 1.12)	1.541 (1.10 ; 2.15)*
Psychological distress (Mean)	1.010 (0.99 ; 1.03)	1.010 (0.99 ; 1.02)	1.005 (0.99 ; 1.03)
PCS (Mean)	0.997 (0.98 ; 1.01)	0.997 (0.98 ; 1.01)	0.998 (0.98 ; 1.02)
MCS (Mean)	0.994 (0.98 ; 1.00)	1.002 (0.99 ; 1.01)	0.992 (0.98 ; 1.01)
**Chronic conditions**			
0	Reference	Reference	Reference
1	0.764 (0.61 ; 0.96)*	1.190 (0.95 ; 1.49)	0.894 (0.66 ; 1.21)
2	0.856 (0.62 ; 1.19)	1.029 (0.75 ; 1.42)	0.968 (0.61 ; 1.52)
3 or more	0.873 (0.59 ; 1.30)	0.636 (0.40 ; 1.01)	0.371 (0.19 ; 0.74)**
Baseline	Reference	Reference	Reference
12 month follow-up	0.943 (0.85 ; 1.04)	1.175 (1.03 ; 1.34)*	1.131 (0.97 ; 1.32)

AOR=Adjusted Odds Ratio; CI=Confidence Interval

*** P<0.001

** P<0.01

* P<0.05; PCS=Physical health Component Summary; MCS=Mental health Component Summary

## Discussion

The study found at baseline high rates of daily tobacco use only (24.0%), problem drinking only (19.6%) and concurrent daily tobacco use and problem drinking (9.5%) in this urban population in South Africa. These results seem similar to a previous national population-based survey in South Africa, with 18.2% current tobacco users only, 20.3% problem drinkers only and 9.6% concurrent current tobacco users and problem drinkers [1]. In this longitudinal study over one year, compared to Time 1 problem drinking only significantly increased to Time 2, while concurrent daily tobacco use and problem drinking also increased but not significantly. The increase in problem drinking may be attributed to the identified risk factor of having higher income and thus better purchasing power that may have led to increased alcohol consumption. On the other hand, problem drinking only decreased with age, which is consistent with another study in South Africa [1] and a longitudinal study in USA [16].

Several risk factors (sociodemographic and health factors but not psychological distress, mental and physical problems as well as chronic conditions) were jointly associated with concurrent daily tobacco use and problem drinking, as well as problem drinking or daily tobacco use. Similar to previous studies [1, 17], we found that male gender was strongly associated with daily tobacco use, problem drinking and concurrent daily tobacco use and problem drinking. While older people were more likely than younger people to use tobacco daily, the pattern was reversed for problem drinking, within younger people being more likely to pursue problem drinking. The prevalence of concurrent daily tobacco use and problem drinking was about 6 times higher among male than female respondents. This finding conforms to previous studies in South Africa [1]. In agreement with other studies [18], a lower education level was associated with daily tobacco use.

However, unlike in other studies [3, 4, 17, 18], there was no association between psychological distress, poor physical quality of life and problem drinking and daily tobacco use. We found that problem drinking was highly associated with daily tobacco use and vice versa, similar to other studies [1, 19-22]. The identification of several sociodemographic and health behaviour factors may help in better targeting intervention programmes for this urban dweller population. This study had several limitations. Information in this study was self-reported; hence, subject to bias. Further, the follow-up period in this longitudinal study was only one year and future studies should have follow-up assessments over a longer period.

## Conclusion

We found high prevalences of daily tobacco use, problem drinking and concurrent daily tobacco use and problem drinking among urban dwellers in South Africa. Further research is required for determining appropriate interventions in this study population.

### What is known about this topic

- In the South African National Health and Nutrition Examination Survey, conducted in 2012, of adult South Africans, 9.6% were engaged in concurrent current tobacco use and problem drinking, 20.3% in problem drinking only and 18.2% in current tobacco use only.

### What this study adds

- This study shows in a longitudinal study an increase in the prevalence problem drinking by 1.5% and conjoint alcohol and tobacco use by 0.8%;
- The strong association between daily tobacco use and problem drinking;
- The importance of identifying risk factors for daily tobacco use only, problem drinking only and conjoint daily tobacco use and problem drinking separately in order to make interventions more effective.

## Competing interests

The authors declare no competing interests.
